# Total Knee Arthroplasty Improved Locomotive Syndrome in Knee Osteoarthritis Patients: A Prospective Cohort Study Focused on Total Clinical Decision Limits Stage 3

**DOI:** 10.1155/2021/3919989

**Published:** 2021-07-08

**Authors:** Shigeaki Miyazaki, Saori Yoshinaga, Kurumi Tsuruta, Amy Hombu, Yoshinori Fujii, Hideki Arakawa, Takero Sakamoto, Etsuo Chosa

**Affiliations:** ^1^Rehabilitation Unit, University of Miyazaki Hospital, 5200 Kihara Kiyotake Miyazaki, Miyazaki 889-1692, Japan; ^2^School of Nursing, Faculty of Medicine, University of Miyazaki, 5200 Kihara Kiyotake Miyazaki, Miyazaki 889-1692, Japan; ^3^Center for Language and Cultural Studies, University of Miyazaki, 1-1 Gakuen Kibanadai-nishi, Miyazaki, Miyazaki 889-2192, Japan; ^4^Department of Mathematics Education, Faculty of Education, University of Miyazaki, 1-1 Gakuen Kibanadai-nishi, Miyazaki, Miyazaki 889-2192, Japan; ^5^Department of Orthopaedic Surgery, Faculty of Medicine, University of Miyazaki, 5200 Kihara Kiyotake Miyazaki, Miyazaki 889-1692, Japan

## Abstract

**Purpose:**

The purpose of this study is to investigate the treatment efficacy of total knee arthroplasty (TKA) on locomotive syndrome (LS) focusing on total clinical decision limit (CDL) stage 3 leading to revealing the motor function indicators that can predict LS improvement in knee osteoarthritis patients who had received TKA.

**Methods:**

This prospective cohort study was conducted in 47 patients evaluated as total CDL stage 3 before TKA who received primary TKA on the operated side and were diagnosed with Kellgren-Lawrence grade 2, 3, or 4 knee osteoarthritis on the nonoperated side. LS was evaluated using stand-up test, two-step test, and 25-Question Geriatric Locomotive Function Scale. In addition, the motor function indicators which could predict the LS improvement were examined. All assessments were conducted before TKA and three months after TKA.

**Results:**

Of the 47 subjects who were evaluated to be in total CDL stage 3 before TKA, 13 patients (27.7%) were determined to show improvements in total CDL. From the result of the decision tree analysis, when the CDL of the two-step test before TKA was 1 or less, the improvement rate was 83.3%. Even if the CDL of the two-step test before TKA was higher than 1 and if the 3 m-Timed Up and Go test (3m-TUG) before TKA was 9.6 or less, the improvement rate was 50%.

**Conclusions:**

As of three months after surgery, TKA can improve LS in about 30% of knee osteoarthritis patients. A two-step test before TKA and 3m-TUG before TKA can be used as motor function indicators to predict LS improvement. This study provides useful information for setting the goal for rehabilitation prior to surgery.

## 1. Introduction

Total knee arthroplasty (TKA) is surgical implantation performed to relieve pain and improve daily living activities in patients with knee joint diseases. Patients who underwent this treatment can be expected to regain social wellbeing at an early stage. In a previous study of 5,649 patients, the first 25-30 years of long-term follow-up conducted on TKA reported the implant survival rate of 94.2% at 25 years and 92.4% at 30 years. Thereafter, patients have 38.1 times high risk of experiencing death for various reasons rather than experiencing failing implantation [1]. The current TKA shows to have long-term functioning durability. In addition, TKA and related physical therapy have been reported to reduce pain and improve motor function in many patients [2–5]. Thus, TKA used for therapeutic treatment is increasing worldwide. However, not all patients reported having a high level of satisfaction after TKA. Several studies showed that the level of satisfaction and function in patients after TKA is lower than THA [6, 7].

Locomotive syndrome (LS) is a concept introduced by the Japanese Orthopaedic Association (JOA) in 2007. LS refers to symptoms related to a decline in the locomotor function such as sitting, standing, and walking due to musculoskeletal disorder [8]. When the symptoms progress and impact on daily life, nursing care becomes necessary [8, 9]. LS is caused by locomotor diseases including osteoarthritis, osteoporosis, degenerative spondylosis, spinal canal stenosis due to spondylosis, and sarcopenia [10]. The JOA developed LS risk tests aimed to identify LS patients. The LS risk tests consist of three assessments: stand-up test, two-step test, and 25-Question Geriatric Locomotive Function Scale (GLFS-25) [11]. The JOA also proposed clinical decision limits (CDL) as a guideline for evaluating the risks of LS [12]. In 2015, CDL included two stages: stage 1 for the early signs of declining mobility and stage 2 for progressively declining mobility. After JOA investigated how the LS could be improved and applied in medical treatment, stage 3 was added in 2020. Stage 3 for progressively declining mobility with difficulty in social participation was based on results of studies on the relationship between LS and frailty.

Previous studies reported the treatment efficacy of LS in knee surgeries including TKA, unicompartmental knee arthroplasty, or high tibial osteotomy. According to the results, all 43 patients (100%) with knee joint diseases were total CDL stage 2 before surgery. Among them, 11.6% of patients showed improvement from stage 2 to stage 1 six months after surgery [13]. However, up to date, no studies have examined the treatment efficacy of TKA in patients with total CDL stage 3 knee osteoarthritis.

The purpose of this study is to investigate the treatment efficacy of TKA on LS focusing on total CDL stage 3 leading to revealing the motor function indicators that can predict LS improvement in patients with bilateral knee osteoarthritis who had received primary TKA.

## 2. Methods

### 2.1. Study Design and Statement of Ethics

This prospective cohort study was approved by the Institutional Research Ethics Board of the authors' institution. Informed consent, which was publicly announced on the institution's website, had been obtained from all participants.

### 2.2. Patient Selection

**Figure 1:**
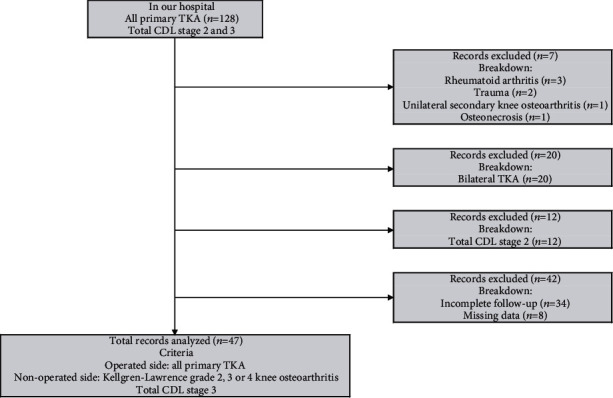
Patient selection flow chart. The subjects in this study were patients who received primary TKA on the operated side and were diagnosed with Kellgren-Lawrence grades 2, 3, or 4 knee osteoarthritis on the nonoperated side with Total CDL stage 3.

The target subjects of this study were patients who received primary TKA on the operated side and were diagnosed with Kellgren-Lawrence grade 2, 3, or 4 knee osteoarthritis on the nonoperated side between October 2018 and January 2021. Among them, patients were selected with total CDL stage 3 in the evaluation before TKA who consented to participate in the evaluations both before and three months after TKA. The followings were excluded from this study: patients with rheumatoid arthritis, trauma, unilateral secondary knee osteoarthritis, and osteonecrosis types of knee joint diseases, patients who received bilateral TKA, patients who were evaluated as total CDL stage 2, and patients with incomplete data sets of outcome measures. After a careful selection and strict screening, there were 47 patients as the subjects of this study (Table 1 and Figure 1).

Among these 47 patients who underwent primary TKA, 26 cases received TKA on the right side and 21 cases on the left side. All these TKA cases were performed by the Department of Orthopaedic in the hospital where the authors are affiliated. The TKA approach was performed with the subvastus approach, and the implant was performed using PS type (posterior stabilized type) that dissected the posterior cruciate ligament. The typical length of hospital stay for TKA was 18 days. The number of rehabilitation days was 15 days, excluding days of hospitalization, surgery, and discharge. Post-TKA rehabilitation started the day after TKA when all subjects were allowed to support their full body weight. During hospitalization, the patients received 60 to 80 minutes of one-on-one rehabilitation twice a day. Outpatients received 20 to 40 minutes of one-on-one rehabilitation two to three times a week. All subjects who underwent rehabilitation for three months after TKA aimed to reduce pain, increase range of motion, obtain normal neuromuscular coordination, reinforce gait patterns, and improve activities of daily life (ADL) by strengthening the muscles and motor functions.

### 2.3. Outcome Measures

#### 2.3.1. Primary Outcome Measures

In this study, LS improvement was evaluated by using the LS risk tests introduced by JOA: the stand-up test, the two-step test, and the GLFS-25 [11]. LS was conducted before and three months after TKA. The results of the CDL for each test and the total CDL were classified as stage 0, 1, 2, or 3.

1. The stand-up test is to assess leg strength. The stools of four different heights 40, 30, 20, and 10 cm in accordance with JOA guidelines were prepared. The subjects were tested by standing up from a sitting position, first with both legs and then with one leg, at each height starting from 40 cm. This test is to quantify leg strength [9]. A nine performance scoring system was adopted [11]: 0 (inability to stand); 1, 2, 3, or 4 (stand using both legs from a height of 40, 30, 20, and 10 cm, respectively); and 5, 6, 7, and 8 (stand using one leg from a height of 40, 30, 20, and 10 cm, respectively). Scores <2, < 3, and< 5 were classified as CDL stages 3, 2, and 1, respectively. The instructions for quantifying motion were (1) fold arms in front of the chest,(2) place feet shoulder-width apart, (3) position lower legs at an angle of approximately 70° to the floor, (4) stand up without gaining momentum, and (5) maintain the standing posture for 3 seconds
2. The two-step test is for measuring stride length. Furthermore, the results of this test can also be used to assess walking ability, including leg strength, balance, and flexibility of the patients. The subjects were tested by taking two steps with the longest possible stride, and then the stride lengths for the two steps were measured. The test score was calculated using the total length of the two steps divided by the subject's height. Scores <0.9, ≥1.1 to <1.3, and ≥ 0.9 to <1.1 were classified as CDL stages 3, 2, and 1, respectively. The instructions for quantifying motion were (1) stand with the tiptoes of both feet aligned at the starting line, 2) take two long strides without losing balance, (3) align tiptoes of both feet when stopping, (4) repeat if out of balance, (5) do not jump, and (6) do the test twice and adopt the better score
3. GLFS-25, a self-assess questionnaire of 25 questions, was used for assessing the physical status and living circumstances. This test measures the physical pain and activities of daily life over the past month prior to the test. Scores ≥24 points, ≥16 to <24 points, and ≥ 7 to <16 points were classified as CDL stages 3, 2, and 1, respectively
4. The total CDL is determined based on the results of the tests mentioned above, namely the stand-up test, two-step test, and GLFS-25. The stage from each of the tests that showed the mobility function has decreased the most was used to classify the patient's final total CDL outcome for analysis

#### 2.3.2. Secondary Outcome Measures

Electronic equipment was used for measuring quiet standing posture and walking movement: optical motion capture system (Vicon Nexus 2.10, Vicon Motion Systems, London, UK) with 13 infrared cameras (MX T20-S and Vantage 8, Vicon Motion Systems) and six force plates (OR6-5 and BP400600, Advanced Mechanical Technology, Inc., Watertown, MA, USA). The sampling frequency was set at 100 Hz, and all the equipment took the measurements synchronously. The subjects were required to wear skin-tight clothes (Under Armour, Baltimore, MD, USA). Thirty-five, 14 mm diameter, reflective markers were attached to the subjects according to the plug-in-gait model protocol [14, 15]. All outcome measures were taken both before and three months after TKA.

*(1) Mean Weight-Bearing Ratio of the Quiet Standing Posture*. Quiet standing posture was measured for five seconds using two force plates. The mean weight-bearing ratio (the mean load on the operated side divided by the mean load on the unoperated side) was calculated from the data for the measured quiet standing posture using analysis software (Vicon Nexus 2.10). The instructions for quantifying posture were (1) place feet shoulder-width apart, (2) position the upper limbs at an angle of approximately 30° to the truck, and (3) maintain the standing posture for 5 seconds.

*(2) Temporospatial Parameters in Walking*. The patients were asked to practice walking barefoot freely on a floor force plate. Measurements were taken three times when patients could walk without feeling uncomfortable. The data from the movement which the subjects were most satisfied with were adopted. The analysis software (Vicon Nexus 2.10) was used to calculate the cadence, stride time, step time, single support time, double support time, stride length, step length, and walking speed on the operated side of the knees. In addition, presentation software (Polygon 4.3) was used to standardize one gait cycle as 100%. The instructions for quantifying motion were (1) walk freely and (2) take a 20-second rest between motions.

*(3) 3 m-Timed up and Go Test (3m-TUG)*. The subjects were asked to stand up from a sitting position on a chair, walk to a marked spot of 3 meters away, return to the chair, and sit down. The time taken for these actions was measured twice, and the shorter one was adopted for analysis [16]. The instructions for quantifying motion were (1) place hands on the thigh while leaning lightly on the back at the starting position, (2) follow the call of the measuring person to perform a series of “maximum walking speed” actions, and (3) do the test twice and adopt the better score.

### 2.4. Statistical Analysis

The sample size was determined by the number of patients with knee diseases who received primary TKA in our hospital during the study period and excluded those who did not meet the criteria of patient selection. Total CDL stage 3 indicates a very high risk of losing mobility and the inability to live independently. This study examined the efficacy of TKA on LS in patients with total CDL stage 3 and motor function indicators that predict LS improvement. All data were expressed as means ± standard deviation (SD). The distribution of each CDL stage ratio for the three LS tests and the total CDL stage was performed using Wilcoxon's signed-rank test. The paired *t*-test was applied to examine the changes in various LS test scores and each motor functional parameter between before TKA and three months after TKA groups. The unpaired *t*-test was applied to examine the differences in age, body mass index (BMI), preoperative various LS test score, each of the preoperative motor functional parameter between the two groups, and improvement and nonimprovement groups, which were divided based on the improvement of the total CDL stage before TKA to three months after TKA. Stepwise multiple logistic regression and decision tree analyses were performed to determine the most important preoperative factors of improvement in total CDL stage. All statistical analyses were performed with a significance threshold set at *p* < .05 using IBM SPSS 27.0 (IBM Corp., Released 2020. Armonk, NY, USA).

## 3. Results

The follow-up time for all 47 subjects was conducted for a duration of 3 months after TKA. The distribution of the CDL stage for the three LS tests and the total CDL stage for all subjects are shown in Table 2. There was a significant change in the percentage of subjects across all four CDL stages: GLFS-25 and total CDL stage, from before TKA to three months after TKA (*p* < 0.001). At three months, the percentage of subjects with total CDL stage 3 decreased, with a concomitant increase in the percentage of subjects with total CDL stages 2, 1, and 0, with the same change in grade distribution for each of the three tests. There was no significant change observed in the stand-up test and two-step test from before TKA to three months after TKA.

Table 3 shows the change in the values of each test before and after TKA. The GLFS-25, weight-bearing ratio (*p* < .001), single support time, step length (*p* < .01), 3m-TUG, double support time, and walking speed (*p* < .05) showed significant improvements.

Table 4 shows the difference in each parameter between the improvement group and the nonimprovement group. There were significantly higher values in the before TKA improvement group observed in the two-step test (*p* < .01), cadence, and walking speed (*p* < .05). The results of the stepwise multiple logistic regression analysis demonstrated that the preoperative two-step test (*p* < .01) and preoperative single support time (*p* < .05) were significant factors associated with the total CDL stage improvement (Table 5).

**Figure 2:**
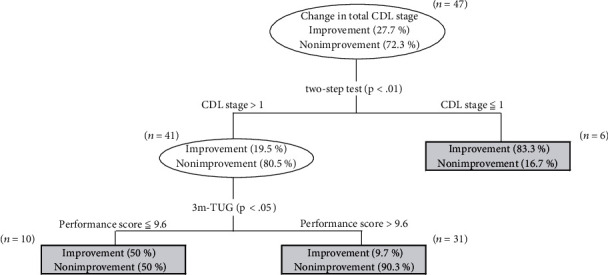
Decision tree analysis. Decision tree analysis was performed to identify the most important preoperative factors related to improvement in total CDL stage.

A decision tree was applied to determine the most important preoperative factors of improvement in the total CDL stage. The decision tree was categorized into 2 branches. Based on the preoperative factors, two-step test, and 3m-TUG, the branches were further divided into three subgroups as shown in Figure 2. The overall LS improvement rate was 27.7%. The preoperative two-step test was determined to be the most relevant factor to the improvement of total CDL stage. In the preoperative two-step test, in the case of CDL stage 1or less group, the improvement of total CDL was 83.3% (5 out of 6 cases); in the case of CDL stages 2 and 3, the improvement of total CDL was 19.5% (8 out of 41 cases). In the subgroup of CDL stages 2 and 3, 3m-TUG was extracted as an improvement factor for the two subgroups. In the subgroup of 9.6 seconds or less, the improvement of total CDL was 50.0% (5 out of 10 cases), whereas in the subgroup of greater than 9.6 seconds, the improvement of total CDL was 9.7% (3 out of 31 cases).

## 4. Discussion

The subjects of this study were patients with bilateral knee osteoarthritis who underwent primary TKA and were evaluated as total CDL stage 3 before TKA. We investigated the effectiveness of LS in total CDL, quiet standing posture, and walking movement before TKA and three months after TKA. In addition, we established the motor function indicators which can predict LS improvement. With the focus on total CDL stage 3, this study is the first, as of this writing, to examine the therapeutic effectiveness of TKA on LS improvement in patients with bilateral knee osteoarthritis and to identify motor function indicators. The most important finding of this study was that of the 47 patients who underwent TKA, at three months after TKA, 27.7% of patients improved from total CDL stage 3 to stage 2 or 1. The two-step test and the 3m-TUG before TKA assessment can be used as indicators to predict the improvement of total CDL.

TKA is a widely accepted treatment because of its noted effectiveness to relieve pain so that patients are able to resume their normal life in society. Therefore, it is important to clarify the extent of improvements made in terms of total CDL after TKA. In a previous cohort study, examining the effects of the operation in patients with knee joint surgery including TKA who were total CDL stage 2 before surgery with LS in a progressive state, it was reported that 11.6% of patients improved in total CDL stage six months after the operation and 9.3% twelve months after the operation [13]. However, to date, there are no reports on the efficacy of treatment in knee osteoarthritis patients focusing on CDL at three months after TKA. Generally speaking, bilateral knee osteoarthritis is the most common type of osteoarthritis, and most patients undergoing TKA have total CDL stage 3 before TKA. Based on our clinical experiences, there were many cases in which ADL improved in a short period by applying unilateral TKA. Thus, in this study, we hypothesized that applying unilateral TKA to patients with bilateral knee osteoarthritis would have a positive effect on the unoperated side and improve LS even in a short period of three months. As a result, this study showed a 27.7% improvement in stage 3, the maximum stage of total CDL, despite the short-term assessment of merely three months after TKA. Our study demonstrated that LS in the short-term at three months after surgery is of considerable clinical significance. Consequently, even with the conditions of decline in the motor function or hindrance in social participation, TKA is a surgical treatment that is expected to be effective within a short period.

The LS risk test showed significant improvement in the GLFS-25, but no significant improvement in the stand-up test and two-step test. The GLFS-25 was developed as a screening tool for elderly patients with motor dysfunction; it evaluates the degree of difficulty to carry out daily activities in the last month before TKA [17, 18]. Before TKA, the patients with CDL stage 3 had difficulties in social participation, and their GLFS-25 score was in a high level of 40.6 points. The results indicated that the patients had high level of difficulty in daily life before TKA. In three months after TKA, the score dropped to 25.5 points which indicated a significant improvement that made it easier for social participation. In addition, there was no significant improvement in the stand-up test and the two-step test. The only significant improvement was observed in GLFS-25 indicated the subjective evaluation was improved after TKA. The stand-up test was report to show no CDL improvement in the knee joint surgery including TKA one year after surgery [13]. The standing motion requires a proper joint range of motion, flexibility, and balance in addition to lower limb strength [9, 11]. Specifically, knee extension muscular strength centering around the hip joint, knee joint range of motion, and quadriceps femoris is important. A systematic review of lower limb strength after TKA by Schache et al. [19] reported that despite the emphasis on strengthening the quadriceps muscle in most rehabilitation protocols, patients showed persistently weaken quadriceps muscle strength compared to the patients in the control group. This suggests that the alleviation of knee joint pain contributes to the recovery of the motor function, but not so much to improving the weakening of the quadriceps muscles.

3m-TUG has been widely used to assess the motor function in the elderly because it is highly related to daily living functions such as lower limb muscle strength, balance, walking ability, and susceptibility to falls [16]. Gait movement tends to become impaired with age. The parameter is shown as follows: walking independently 3m − TUG < 10 seconds, nearly independent at 11–19 seconds, unstable at 20–29 seconds, and impaired at ≥30 seconds. The results of this study showed that walking speed improved significantly from 13.5 seconds before TKA to 10.8 seconds three months after TKA which suggests that patients could recover to walk almost independently three months after TKA. The mean weight-bearing ratio in the quiet standing posture improved significantly from 0.9 before TKA and 1.1 at three months after TKA. This indicates that the weight-bearing ratio increased on the operated side which was high on the unoperated side before TKA, but three months after TKA, the ratio became almost equal on both operated and unoperated sides. Compared with before TKA, walking speed improved significantly three months after TKA in the temporospatial parameters in walking that was influenced by prolonged single support time and shortened double support time of the increasing weight-bearing ratio on the operated side. According to Yoshida et al. [20], there were concomitant symmetrical improvements in temporospatial and kinetic parameters in walking as interlimb differences in quadriceps muscle strength decreased after TKA. As a result, there were significant improvements in TUG, stair-climbing test, knee range of motion, strength, and vertical ground reaction force between three months and one year after TKA due to the decrease in interlimb differences in quadricep muscle strength. In addition, the improvement in biomechanical symmetry due to the decrease in the interlimb differences is attributed to the muscle weakness on the unoperated side. Thus, at three months after TKA, improvements in posture and walking ability were demonstrated in parallel with improvements in mobility. These improvements accompanied by the decrease in interlimb differences in the quadriceps muscle strength may continue beyond three months after TKA.

A discrepancy in the motor function assessment parameters was found in the before TKA parameters between the total CDL improvement group and the nonimprovement group. In particular, it was considered that the higher the function before TKA, the better improvement can be expected in the two-step test, cadence of operated side, and walking speed. Therefore, the present study examined the motor function indicators that influence the improvement of LS. A stepwise multiple logistic regression analysis was performed for each parameter. Before TKA, CDL of the two-step test and single support time of the operated side were found to be significantly related to improvements in total CDL. A decision tree was established to identify the characteristics of subjects who were expected to show improvements from the groups with different improvement rates. As a result, three groups with different improvement rates were identified. When the CDL was ≤1, the improvement rate was 83.3% in the CDL of the two-step test before TKA. Even if the CDL was >1 in the CDL of the two-step test before TKA, if the 3m-TUG was ≤9.6, the improvement rate was 50%. On the other hand, if the CDL was >1 in the CDL of the two-step test before TKA and the 3m-TUG was >9.6, the improvement rate was the lowest at 9.7%. Therefore, it can be determined that the two-step test and the 3m-TUG before TKA can be motor function indicators to identify the effects of LS improvements. The two-step test as well as the 3m-TUG can be used primarily as motor function assessment to estimate walking ability. Maintaining the walking function as much as possible before TKA is considered effective in LS improvement after TKA. Yamaguchi et al. [21] reported that more than 40% of patients who had increasing pain due to knee osteoarthritis at the one-year follow-up were unable to walk at the seven-year follow-up. They suggested that the patients with knee osteoarthritis whose pain worsened within one year should receive early intervention such as elective surgery or rehabilitation to prevent future loss of the walking function. Early intervention to maintain the walking function is beneficial for patients with knee osteoarthritis. However, to date, no motor function indicators are used to predict LS improvements in patients with TKA. The results of this study provide useful information for considering when to conduct TKA in order not to miss the “windows of opportunity.” Furthermore, the evaluation of the two-step test and the 3m-TUG before TKA make it possible to predict functional improvement after TKA. This study is of great clinical significance in providing useful information for setting the goal of rehabilitation prior to implementation.

There were two limitations of this study. First, the sample size of our data was small. Since the sample size is small, the result of stepwise multiple logistic regression analysis may be biased. However, the purpose of this study was not to evaluate the performance of the artificial joint (or implant survival rate, etc.) but to evaluate the degree of the motor function recovery at three months after TKA using the LS risk test. Second, the knee joint range and the knee joint score have not been examined for any association with the LS risk test. The total score of the range of motion and muscle strength was evaluated by the LS risk test. Further research is needed to investigate these important factors.

## 5. Conclusions

Among the 47 patients with bilateral knee osteoarthritis who underwent primary TKA, at three months after TKA, 27.7% of the patients made an improvement from total CDL stage 3 to stage 2 or 1. The two-step test before TKA and the 3m-TUG before TKA were identified to be the main motor function indicators for predicting the improvement of total CDL. Making efforts to maintain the walking function before TKA is considered effective in improving LS after TKA. This information will be useful in setting goals for rehabilitation before surgery.

## Figures and Tables

**Table 1:** Subjects' characteristics.

Characteristic	Total (*n* = 47)
Age (y)	74.6 ± 7.3
Sex (male)	12
Height (cm)	150.5 ± 8.6
Weight (kg)	62.6 ± 11.5
Body mass index (BMI) (kg/m^2^)	27.6 ± 4.2

Age, height, weight, and BMI values are means ± standard deviation.

**Table 2:** Distribution of the CDL stage for the three LS tests and the total CDL stage.

		Stage 0		Stage 1		Stage 2		Stage 3		Wilcoxon signed-rank test
		*N*	%	*N*	%	*N*	%	*N*	%	*p* value
Stand-up test	Before TKA	0	0	8	17.0	26	55.3	13	27.7	0.317
	Three months after TKA	2	4.3	6	12.8	29	61.7	10	21.3	
Two-step test	Before TKA	0	0	6	12.8	16	34.0	25	53.2	0.216
	Three months after TKA	0	0	7	14.9	20	42.6	20	42.6	
GLFS-25	Before TKA	0	0	0	0	3	6.4	44	93.6	< 0.001^∗∗∗^
	Three months after TKA	4	8.5	10	21.3	9	19.1	24	51.1	
Total CDL stage	Before TKA	0	0	0	0	0	0	47	100	< 0.001^∗∗∗^
	Three months after TKA	0	0	1	2.1	12	25.5	34	72.3	

Significantly different: ^∗∗∗^*p* < .001.

**Table 3:** Changes in the three LS test scores and each functional parameter from before to three months after TKA.

	Before TKA	Three months after TKA	Paired *t*-test *p* value
Stand-up test	1.92 ± 0.72	1.98 ± 0.74	0.519
Two-step test	0.85 ± 0.20	0.91 ± 0.20	0.073
GLFS-25	40.57 ± 13.12	25.53 ± 17.89	< 0.001^∗∗∗^
3m-TUG	13.49 ± 9.51	10.83 ± 3.21	0.038^∗^
Weight-bearing ratio	0.90 ± 0.32	1.11 ± 0.39	< 0.001^∗∗∗^
Cadence (operated side)	104.1 ± 19.8	108.5 ± 14.3	0.066
Stride time (operated side)	1.21 ± 0.36	1.13 ± 0.17	0.077
Step time (operated side)	0.61 ± 0.19	0.56 ± 0.11	0.056
Single support time (operated side)	0.37 ± 0.07	0.41 ± 0.06	0.004^∗∗^
Double support time (operated side)	0.42 ± 0.28	0.32 ± 0.11	0.015^∗^
Stride length (operated side)	0.75 ± 0.21	0.80 ± 0.17	0.070
Step length (operated side)	0.38 ± 0.12	0.42 ± 0.09	0.005^∗∗^
Walking speed	0.67 ± 0.26	0.73 ± 0.18	0.031^∗^

Significantly different: ^∗^*p* < .05, ^∗∗^*p* < .01, ^∗∗∗^*p* < .001.

**Table 4:** Differences in the preoperative test scores and various preoperative functional parameters between the improvement group and the nonimprovement group.

		Improvement group (*n* = 13)	Nonimprovement group (*n* = 34)	Unpaired *t*-test *p* value
	Age (y)	74.3 ± 7.3	74.7 ± 7.4	0.859
	BMI (kg/m^2^)	28.2 ± 4.1	27.3 ± 4.3	0.525
Before TKA	Stand-up test	2.08 ± 0.49	1.85 ± 0.78	0.251
	Two-step test	0.99 ± 0.15	0.80 ± 0.19	0.002∗∗
	GLFS-25	34.85 ± 11.16	42.77 ± 13.30	0.064
	3m-TUG	9.75 ± 2.19	14.92 ± 10.81	0.096
	Weight-bearing ratio	0.81 ± 0.36	0.94 ± 0.30	0.208
	Cadence (operated side)	115.3 ± 15.5	99.8 ± 19.8	0.014∗
	Stride time (operated side)	1.06 ± 0.15	1.27 ± 0.40	0.068
	Step time (operated side)	0.55 ± 0.10	0.63 ± 0.21	0.167
	Single support time (operated side)	0.35 ± 0.07	0.39 ± 0.07	0.111
	Double support time (operated side)	0.31 ± 0.11	0.46 ± 0.32	0.108
	Stride length (operated side)	0.82 ± 0.23	0.73 ± 0.20	0.184
	Step length (operated side)	0.43 ± 0.11	0.36 ± 0.11	0.076
	Walking speed	0.80 ± 0.29	0.61 ± 0.23	0.023^∗^

Significantly different: ^∗^*p* < .05, ^∗∗^*p* < .01.

**Table 5:** Stepwise multiple logistic regression analysis between improvement effects of total CDL stage and various locomotive tests before TKA.

Variable	*B*	Exp (*B*)	95% CI	*p* value
Two-step test (CDL stages 0, 1, 2, and 3)	-1.978	0.138	0.038-0.505	0.003^∗∗^
Single support time (operated side)	-12.312	0.000	0.000-0.735	0.044^∗^
Constant	8.046			
Nagelkerke *R*^2^	0.416			

Significantly different: ^∗^*p* < .05, ^∗∗^*p* < .01.

## Data Availability

The data used to support the findings of this study are included within the article.
